# Correction: Motivating factors for physical activity participation among individuals with chronic obstructive pulmonary disease: A qualitative study applying the motivation, opportunity, and ability model

**DOI:** 10.1371/journal.pone.0306494

**Published:** 2024-06-27

**Authors:** Yuanyu Liao, Jiaohua Yu, Yuxin Zhan, Yunfang Liu, Yaoling Zhou, Huan Wang, Xinghong Liu, Weiwei Wang, Yu Ma, Fenfen Lan

There are errors in the author affiliations. The correct affiliations are as follows:

Yuanyu Liao^1, 2^, Jiaohua Yu^1^, Yuxin Zhan^3^, Yunfang Liu^4^, Yaoling Zhou^2, 5^, Huan Wang^4^, Xinghong Liu^6^, Weiwei Wang^2^, Yu Ma^2^, Fenfen Lan^2^

**1** Department of Nursing, Union Hospital, Tongji Medical College, Huazhong University of Science and Technology, Wuhan, Hubei, China, **2** School of Nursing, Tongji Medical College, Huazhong University of Science and Technology, Wuhan, Hubei, China, **3** Department of Neurosurgery, Union Hospital, Tongji Medical College, Huazhong University of Science and Technology, Wuhan, Hubei, China, **4** Department of Thoracic Surgery, Union Hospital, Tongji Medical College, Huazhong University of Science and Technology, Wuhan, Hubei, China, **5** School of Nursing, Huanggang Polytechnic College, Huanggang, Hubei, China, **6** Department of Cardiovascular Surgery, Union Hospital, Tongji Medical College, Huazhong University of Science and Technology, Wuhan, Hubei, China.
