# Supplementary material for: Motivating factors for physical activity participation among individuals with chronic obstructive pulmonary disease: A qualitative study applying the motivation, opportunity, and ability model
Source: PLoS One. 2024 May 23;19(5):e0303858. doi: 10.1371/journal.pone.0303858 (PMC11115224; doi:10.1371/journal.pone.0303858)
Supplement: S2 Appendix — (DOCX) [file pone.0303858.s002.docx]

**S2 Appendix. Interview guide**

**Note:** while this guide provides instructions on the types of questions to ask, semi-structured interviews remain open-ended, allowing for the emergence of new ideas.

- Can you explain your understanding of "physical activity"? (Prompts: What do you think physical activity includes? How do you perceive its benefits for your health?)
- What motivates you to engage in physical activities? (Prompts: What drives your participation in physical activities? How do you perceive and experience engaging in physical activities? Do you enjoy these activities? How do they impact your feelings?)
- What factors do you believe contribute to your consistent involvement in these activities? (Prompts: For instance, family support, establishing a routine?)
- What obstacles have hindered your participation in these activities? (Prompts: For example, fear of breathlessness, complications?)
- Can you describe your exercise routine before you became ill?
- What changes have occurred in your physical activity since you became ill? What reasons led to these changes?
- Are you content with your current activity level? What factors contribute to this satisfaction? Could you elaborate further on this?
- What kinds of physical activities are you typically engaged in? (Prompts: Could you describe the physical activities you currently participate in? For instance, their intensity, type, duration, frequency, goals, and outcomes.)
- What challenges have you faced during your activities? How do you adapt your activities based on your physical condition? (Prompts: When faced with difficulties during activities, how do you manage and persist?)
- What kind of support do you receive during your activities? (Prompts: Do you have family or friends accompanying you? Do you require assistance from family, peers, or healthcare professionals?)
- Through which channels do you acquire information about physical activity? How do you assess the quality of the information you receive?
- While you're in the hospital, do you feel inclined to receive more education about physical activities? Do you still feel the need for it once you're home after discharge?
- What do you believe can assist both yourself and others in being more active?
- Do you have any additional thoughts to add to this discussion?
- If someone has stopped engaging in activities: Why did you discontinue your activities? What obstacles prevented your participation? How do you think you could become more active again?
