# Supplementary material for: Motivating factors for physical activity participation among individuals with chronic obstructive pulmonary disease: A qualitative study applying the motivation, opportunity, and ability model
Source: PLoS One. 2024 May 23;19(5):e0303858. doi: 10.1371/journal.pone.0303858 (PMC11115224; doi:10.1371/journal.pone.0303858)
Supplement: Attachment — Submitted filename: Response to Reviewers.docx [file pone.0303858.s003.docx]

Yuanyu Liao

Nursing Department, Tongji Medical College Affiliated Union Hospital, Huazhong University of Science and Technology, Wuhan, Hubei Province, China, 430022

School of Nursing, Tongji Medical College, Huazhong University of Science and Technology, Wuhan, Hubei Province, China, 430030

Katrina9820@126.com

February 21, 2024

Subject: **Response to Reviewers for Manuscript PONE-D-24-00066**

Dear Dr. Petra Czarniak,

We would like to express our gratitude to you and the reviewers, Dr. Sarah Markham and James P. Gavin, for providing valuable feedback on our manuscript titled "Motivating Factors for Physical Activity Participation among Individuals with Chronic Obstructive Pulmonary Disease: A Qualitative Study Applying the Motivation, Opportunity, and Ability Model." We appreciate the time and effort invested in evaluating our work and offering constructive suggestions for improvement. We have carefully considered each comment and have made the necessary revisions to address the concerns raised. Below, we provide a detailed response to each reviewer's comments:

**Reviewer: PLOS ONE**

[Reviewer's Comment 1]

Response: We have diligently adhered to the style templates provided by the editors to ensure that the manuscripts meet PLOS ONE's style and file naming requirements. Additionally, the table files have been uploaded to the Pre-submission Analysis and Conversion Engine (PACE) digital diagnostic tool, aligning with PLOS's specifications. Please refer to Table 1 for details.

[Reviewer's Comment 2]

Response: The corresponding author of this article has provided and validated the ORCID iD in Editorial Manager. The ORCID iD is 0009-0008-9002-5370.

**Reviewer #1: Dr. Sarah Markham**

[Reviewer's Comment]

Response: We sincerely appreciate your thoughtful and encouraging assessment of our manuscript titled "Motivating Factors for Physical Activity Participation among Individuals with Chronic Obstructive Pulmonary Disease: A Qualitative Study Applying the Motivation, Opportunity, and Ability Model." Your acknowledgment of the study as an interesting, well-executed, and clinically significant research endeavor is truly gratifying.

We are thrilled that you recognize the potential global impact of our findings and their applicability to real-world clinical practices. Your positive evaluation serves as motivation to continue our efforts in contributing valuable insights to the field of chronic obstructive pulmonary disease management.

We are grateful for the absence of specific revision suggestions, indicating that our manuscript aligns well with the standards of PLOS ONE. Your feedback encourages us to maintain the rigor and relevance of our research, and we are committed to ensuring the clarity and coherence of our work.

Once again, we express our gratitude for your positive appraisal and your time devoted to reviewing our manuscript. We look forward to the possibility of contributing to the dissemination of knowledge in PLOS ONE.

**Reviewer #2: James P. Gavin**

[Reviewer's Comment 1: Description of participants in the Methods section of the abstract should include both inpatient and outpatient individuals]

Response: We appreciate Dr. Gavin's feedback on the need for a detailed description of participants from different sources in the Methods section of the abstract. In response to this comment, we have supplemented this section to align with the overall manuscript. Please refer to the revised manuscript on page 2, lines 28-29.

[Reviewer's Comment 2: Introduction should address the maintenance of physical activity]

Response: Regarding the suggestion to incorporate references in the introduction regarding the maintenance of physical activity, including in long-term patients, we have carefully revised the introduction to address this issue. Please see the revised manuscript on page 4, line 82, and page 5, lines 83-86. These modifications enhance the overall coherence and readability of the manuscript.

[Reviewer's Comment 3: The introduction should evaluate the reasons for choosing the MOA model from the perspective of other existing models]

Response: We appreciate Dr. Gavin's insightful suggestions for reexamining and revising other existing models and the MOA model. In response to this suggestion, we have added paragraphs to the introduction section in the revised manuscript to address this issue. Please see the revised manuscript on page 5, lines 95-105, and page 6, lines 106-116. These changes improve the clarity and rigor of our study.

[Reviewer's Comment 4: Adjustments in the Materials and Methods section regarding paragraph order and content addition]

Response: The subsection "Study Design 2.2" has been adjusted to precede "2.1 Research Team and Reflexivity." Regarding the concern about how researchers establish relationships with participants, especially when interactions seem limited to healthcare providers, detailed explanations have been provided in the revised manuscript on page 9, lines 162-172, and page 10, lines 173-174. The title of subsection 2.2.1 has been modified to "Participant Selection and Recruitment." Percentage data for inpatient and within 6 months post-discharge participants have been added to Table 1 on page 8. Two references supporting methods for assessing data saturation have been included in the data collection subsection on page 9, lines 156-157. The data analysis section in the revised manuscript on page 10, lines 176-190, now includes author initials and highlights researchers' involvement in specific steps of the Colaizzi method. We believe these revisions strengthen the validity and reliability of our study results.

[Reviewer's Comment 5: Balance between citations and narrative in the Results section, differentiate between inpatient and post-discharge community residents]

Response: We have carefully reviewed our Results section and made necessary adjustments, including reducing citations, increasing narrative content for a balanced presentation, and explicitly noting which citations are from inpatient versus post-discharge community residents. Relevant concise demographic data (e.g., Participant 17, male, 45 years old) have been added to citations. We have also examined and corrected descriptions that simultaneously state quotes from two participants. Please refer to the revised manuscript on pages 11-19.

[Reviewer's Comment 6: Add content and reflection to the Discussion and Limitations sections]

Response: In the revised Discussion section, we have added content on maintaining physical exercise (page 22, lines 453-455), connecting the MOA model with behavior change theories and future work (page 23, lines 463-469), and highlighting differences in experiences between inpatient and post-discharge/community-residing participants (page 20, lines 393-403). In the revised Limitations section, we have included reflections and comments on the MOA model (page 23, lines 478-481) and limitations related to the study population (page 23, lines 472-476).

In conclusion, we believe that the revisions made in response to the reviewers' comments have significantly improved the quality and clarity of our manuscript. We are grateful for the opportunity to address these valuable suggestions and are confident that the revised manuscript meets the standards of PLOS ONE. We appreciate your consideration of our work and look forward to the opportunity for publication.

Thank you once again for your time and consideration.

Best regards,

Yuanyu Liao

Master's research student

e-mail: Katrina9820@126.com
