# Supplementary material for: Motivating factors for physical activity participation among individuals with chronic obstructive pulmonary disease: A qualitative study applying the motivation, opportunity, and ability model
Source: PLoS One. 2024 May 23;19(5):e0303858. doi: 10.1371/journal.pone.0303858 (PMC11115224; doi:10.1371/journal.pone.0303858)
Supplement: Attachment — Submitted filename: Response to Reviewers.docx [file pone.0303858.s004.docx]

Subject: **Response to Reviewers for Manuscript PONE-D-24-00066R1**

Dear Editor and Reviewers,

We would like to express our gratitude for your insightful comments and suggestions on our manuscript, "Motivating factors for physical activity participation among individuals with chronic obstructive pulmonary disease: a qualitative study applying the motivation, opportunity, and ability model." We have carefully considered each point and made the following revisions:

1. Acknowledgment of Funding: We have added a second grant information: the Hubei Provincial Department of Finance 2024 Research Program (08.01.24011) is also held by JY, the corresponding author of this paper, and a detailed financial disclosure statement was made in the cover letter.

2. Language and Formatting: We undertook a thorough review and revision of grammar, presentation, and formatting throughout the manuscript, particularly new additions to improve clarity and correctness. This included further revisions to the abstract; harmonizing the abbreviations for "chronic obstructive pulmonary disease," "physical activity," and "motivation-opportunity-ability;" and ensuring consistency in the formatting of the authors' names throughout the text, including the added theoretical description paragraph in the Introduction section and the researcher's initials in the Methods section. In addition, we refined the concluding sentences in the Discussion section.

3. Table Revision: We have revised "Occupation" to "Occupational Status" in Table 1, adjusted the legends to meet PLOS ONE formatting requirements, and re-uploaded them to the PACE digital diagnostic tool to ensure compliance.

4. Consistency in Themes: We have ensured consistency between the themes presented in the Results section and the descriptions provided in the abstract.

5. Reference formatting: All references were reviewed and corrected to comply with PLOS ONE guidelines, including formatting adjustments such as replacing commas with semicolons after the year of publication, deleting specific months and days, and deleting redundant spaces. We also completed missing page numbers for references 2, 3, 5, and 16; reconfirmed the absence of doi numbers in article 6, providing PMIDs only; added author names for references 8 and 9, made appropriate adjustments to reference 12, abbreviated journal names for reference 25, and ensured that references 1, 5, 10-17, 21, 24-27, 29, 32, 34, 36, 40, 42, 44-46, 48, 50, 52, 54, 57-58, 60-61 were adjusted to lower case except for initial letters.

6. Update of Supplementary Material: We have updated Appendix S1 (the Consolidated Criteria for Reporting Qualitative Research [COREQ]) to reflect all corresponding modifications.

7.Protocols.io Submission: Since we have provided a comprehensive research methodology in our paper, we confirm that it does not apply to submissions to protocols.io.

Once again, we sincerely appreciate the time and effort invested in reviewing our paper. We believe that the revisions we have made address all the concerns raised by the editor and reviewers, and we are confident that these revisions have significantly improved the quality and clarity of our manuscript.

Thank you for your thorough assessment, and we kindly request that you consider our revised manuscript for acceptance into PLOS ONE. We remain at your disposal for any further clarifications or modifications if necessary.

Sincerely,

Yuanyu Liao

First Author

e-mail: Katrina9820@126.com
