# Supplementary material for: Motivating factors for physical activity participation among individuals with chronic obstructive pulmonary disease: A qualitative study applying the motivation, opportunity, and ability model
Source: PLoS One. 2024 May 23;19(5):e0303858. doi: 10.1371/journal.pone.0303858 (PMC11115224; doi:10.1371/journal.pone.0303858)
Supplement: Attachment — Submitted filename: Response to Reviewers.docx [file pone.0303858.s005.docx]

Subject: **Reply to Reviewer's Comment for Article ID PONE-D-24-00066R2**

Dear Dr. Petra,

We would like to express our gratitude for your insightful comments and suggestions on our manuscript entitled "Motivating factors for physical activity participation among individuals with chronic obstructive pulmonary disease: a qualitative study applying the motivation, opportunity, and ability model" submitted to PLOS ONE. We appreciate the opportunity to address your concerns and improve the quality of our research. Below, we provide a detailed response to each of your points:

Discrepancy between Manuscript and Revised Manuscript with Track Changes: We sincerely apologize for any inconsistencies between the Manuscript and the Revised Manuscript with Track Changes. Upon thorough review, we discovered that the discrepancies arose from the automatic formatting changes that occurred when our Word document was uploaded to the system and converted into a PDF file. For instance, in line 83 of our original Word document, we referenced the study by Dragnich et al., which unfortunately did not appear as [11] after uploading. To mitigate such issues in the future, we have diligently cross-checked both the manuscript in Word format and the system-generated PDF version to ensure that all references are properly cited and that there are no discrepancies between the manuscript and the tracked changes document.

Grammar Adjustments throughout the Manuscript: In response to your suggestions, we have made several grammatical adjustments throughout the manuscript to enhance clarity and accuracy. Specifically, we have rewritten and polished lines 265/266 and 372/373 to better articulate our analysis of the interview data. Additionally, we have reviewed and revised lines 267/268 to maintain logical consistency and prevent grammatical errors. We have also refined lines 277/278 to accurately convey the concept of "oldest-old individuals." Furthermore, we have polished lines 307/308, 315/316, 334/336, and 370/371 to provide a clearer expression of the interviewees' statements.

We believe that these revisions have strengthened the overall quality and academic rigor of our manuscript. Thank you once again for your invaluable feedback and guidance throughout this process. We are confident that the improvements made will contribute to the scholarly excellence of our work.

Sincerely,

Yuanyu Liao

First Author

e-mail: Katrina9820@126.com
