# Supplementary material for: Motivating factors for physical activity participation among individuals with chronic obstructive pulmonary disease: A qualitative study applying the motivation, opportunity, and ability model
Source: PLoS One. 2024 May 23;19(5):e0303858. doi: 10.1371/journal.pone.0303858 (PMC11115224; doi:10.1371/journal.pone.0303858)
Supplement: Attachment — Submitted filename: Response to Reviewers.docx [file pone.0303858.s006.docx]

**Response to Reviewers - Manuscript ID: PONE-D-24-00066R3**

Dear Dr. Petra,

Thank you for your thoughtful review on our manuscript "Motivating factors for physical activity participation among individuals with chronic obstructive pulmonary disease: a qualitative study applying the motivation, opportunity, and ability model," submitted to PLOS ONE (Manuscript ID: PONE-D-24-00066R3). We've addressed your concerns and made the following revisions:

We've updated the COREQ checklist to align with the manuscript. The revised COREQ checklist is attached with tracked changes for clarity.

We've corrected the missing letters in the 9th reference and ensured all references are accurate and meet PLOS ONE standards.

We've tried several times to fix line 191 on page 10 but haven't found a solution yet for the formatting inconsistency post-upload.

Thanks again for your detailed review and helpful suggestions. We trust these revisions improve our manuscript. Please let us know if further adjustments are needed.

Best regards,

Yuanyu Liao

First Author

e-mail: Katrina9820@126.com
